# Pharmacological Separation of Mechanosensory Mechanisms in Rat Urinary Bladder Ex Vivo

**DOI:** 10.1002/prp2.70242

**Published:** 2026-04-01

**Authors:** Igor B. Philyppov, Ganna V. Sotkis, Semen I. Yelyashov, Valeri G. Naidenov, Yaroslav M. Shuba

**Affiliations:** ^1^ Bogomoletz Institute of Physiology of the National Academy of Sciences of Ukraine Kyiv Ukraine

**Keywords:** detrusor smooth muscle, mechanosensitivity, PIEZO1, rat urinary bladder, TREK1, TRPV4, urothelium

## Abstract

The local response of the bladder wall to stretch is believed to result from the coordinated activation of mechanosensitive ion channels located in the plasma membrane of the cells that form the wall's urothelial and detrusor smooth muscle (DSM) layers. While the neuronal bladder control is well defined, the data on the mechanisms of local mechanical sensitivity of the bladder wall are either insufficient or contradictory. The involvement of mechanosensitive ion channels, TREK1, TRPV4, and PIEZO1, in determining stretch‐dependent properties of the rat bladder wall was assessed by tensiometric measurements of stress–strain dependencies of mucosa‐intact and mucosa‐devoid DSM strips combined with channel‐specific pharmacology. Here we show that TREK1, TRPV4, and PIEZO1 are functionally expressed in the rat bladder DSM and urothelium. TREK1 is coupled with bladder wall relaxation via decreasing DSM excitability and releasing of relaxant mediator(s) from the urothelium. TRPV4 in DSM is involved in gradually developing DSM tone during TREK1‐dependent relaxation. Urothelial TRPV4 plays a role in the urothelium‐mediated conversion of mechanical stretch into non‐voiding spontaneous DSM contractions. DSM‐localized PIEZO1 exhibits a higher sensitivity to stretching than TRPV4. Activation of urothelial PIEZO1 leads to the release of the mediator(s) with contractile action on DSM to limit the extent of bladder distention during filling. Our data distinguish the functional involvement of TREK1, TRPV4, and PIEZO1 in the autonomous mechanosensory properties of decentralized urinary bladder wall and provide a strategy for specific pharmacological targeting of bladder contraction/relaxation during different phases of bladder function.

## 1 Introduction

The bladder's failure to respond properly to mechanical stretch may be one of the factors contributing to urinary incontinence, a growing global issue [1]. During filling, the bladder's wall tension gradually increases without triggering neural responses. Once the bladder reaches capacity, mechanical forces in the urothelium and detrusor smooth muscle (DSM) layers activate bladder afferents, causing sensations and initiating parasympathetic signals that lead to DSM contraction and voiding. While the central nervous system's (CNS) neuronal control of the bladder is well documented [2], the exact ways mechanical stimuli affect local detrusor contractility are still not fully understood.

The conversion of mechanical stimuli into electrochemical signals that trigger cellular responses is mediated by mechanosensitive ion channels located in the plasma membrane. Their activity is modulated by local forces arising from membrane stretch or deformation [3]. In the bladder, stretch responses are thought to primarily involve urothelial cells, which release ATP upon mechanical stimulation [4, 5]. This ATP then activates purinergic receptors on primary bladder afferent nerve endings, initiating action potential firing in sensory neurons and transmitting the stretch signal to the CNS for processing and feedback [4]. Direct activation of mechanosensitive suburothelial afferent nerve endings has also been proposed [6]. However, the mechanisms underlying the direct mechanical sensitivity of DSM cells during bladder wall stretching remain poorly understood and sometimes contradictory. Importantly, because the bladder maintains active mechanical responsiveness even after removal from the body, and thus independent of CNS input, the presence of local intrinsic mechanosensory mechanisms is undeniable.

Ex vivo bladder stretch responses are thought to arise from the coordinated activation of mechanosensitive ion channels in both the urothelium and DSM. Among these, the most prominent are cationic, Ca^2+^‐permeable channels TRPV4 [7, 8, 9], PIEZO1 [10, 11], PIEZO2 [10, 12], and the two‐pore‐domain (2P‐domain) K^+^‐selective channel TREK1 [13]. In this study, we investigated the contribution of mechanosensitive ion channels to stretch‐dependent properties of the bladder wall using tensiometric measurements of stress–strain relationships in rat bladder strips, combined with channel‐specific pharmacological interventions. To further evaluate the role of the urothelium, we performed parallel measurements on DSM strips with either an intact mucosal layer or with the mucosa removed.

## 2 Methods

### 2.1 Ethics Statement

All animal protocols complied with the EU Directive 2010/63/EU for animal experiments (http://ec.europa.eu/environment/chemicals/lab_animals/legislation_en.htm) and were approved by the Bogomoletz Institute of Physiology Bioethics Committee (Permission No 1/13 from 15.03.2013).

### 2.2 Preparation of Rat Bladder Strips and Acquisition of Stress–Strain Dependencies

Three‐month‐old male Wistar rats weighing between 200 and 250 g were used in the experiments. The animals were euthanized by exposure to a rising concentration of CO_2_ (fill rate of 50% of the chamber volume per minute), and death was confirmed by subsequent decapitation. The whole urinary bladder was surgically removed and placed in the warmed (37°C), oxygenated (95% O_2_ and 5% CO_2_) Krebs solution (in mM): 120.4 NaCl, 5.9 KCl, 1.2 MgCl_2_, 1.2 NaH_2_PO_4_, 1.8 CaCl_2_, 15.5 NaHCO_3_, 11.5 glucose (рН 7.4).

For detrusor smooth muscle (DSM) strips preparation, the urinary bladder was cut ventrally from the base to the dome and separated into two equal parts. One part was left with intact mucosa, and the other part was mechanically cleaned of mucosa using microsurgical scissors. Both parts were cut into longitudinal strips (diameter ~2 mm, length ~8–10 mm), which were used for tensiometric stress–strain measurements, as detailed elsewhere [14]. Briefly, the strip was positioned in an organ bath (volume 0.5 mL) that was continuously superfused (gravity‐driven flow rate 2 mL/min) with preheated (to 35°C), oxygenated (95% O_2_–5% CO_2_) experimental Krebs solution, with one end of the strip fixed still and another end attached to the capacitative force sensor with the help of ligatures. The distance between the ligature nodes on the strip was 6 mm. The capacitative force sensor was mounted on the micromanipulator. To measure the stress–strain relationship, the strip was stretched every 3.5 min, the time required for 90% relaxation to a new level, by axially moving the micromanipulator with a step of 0.1 mm. The force developed by the strip in response to the stretch was continuously recorded using pCLAMP software and DigiData 1200 on the computer.

Stress is defined as the force per unit area of a material, whereas an axial normal strain is expressed as relative strip lengthening (Δ*l*/*l*_0_, where *l*_0_ is the initial strip length). Thus, the stress–strain relationship was constructed by plotting the force developed by the strip related to the strip cross‐sectional area (i.e., mN/mm^2^) versus axial strain (in %).

The plots of stress–strain dependencies obtained under different conditions and of their ratios were presented at the same X and Y scales to enable direct visual comparison.

### 2.3 Pharmacological Tools

The following pharmacological tools highly selective towards the respective channel to prevent off‐target effects were used in the study: TRPV4 agonist GSK1016790A (Sigma‐Aldrich, G0798) [15] and TRPV4 antagonist HC‐067047 (Sigma‐Aldrich, H4415) [16], PIEZO1 chemical activator Yoda1 (Sigma‐Aldrich, SML1558) [17], and TREK1 inhibitor L‐methionine (Sigma‐Aldrich, M9625) [18, 19]. Nominally Ca^2+^‐free extracellular solution and a non‐specific blocker of mechanogated ion channels, Gd^3+^, were used to appreciate the extent to which the mechanical properties of DSM strips are determined by active mechanosensation and by passive elastic properties of the tissue. Stock solutions of GSK1016790A (10 mM), HC‐067047 (10 mM), and Yoda1 (10 mM) were prepared in DMSO, and stock solutions of L‐methionine (200 mM) and GdCl_3_ (10 mM) were prepared in water. To obtain the working concentration of the compound, an appropriate volume of its stock was added directly to the experimental Krebs solution. The maximum concentration of DMSO in the experimental solutions was 0.1%, which is generally considered acceptable for biological applications (e.g., [20]). The working concentrations of the compounds were adopted from the literature, taking into account the need to achieve near‐saturating effects at the site of action and the higher diffusion barrier in multicellular preparations.

### 2.4 Data Analysis and Statistics

Stress–strain dependencies for isolated DSM strips were obtained from two to three strips from each bladder of at least five animals. First, the control stress–strain dependency was measured on a given strip in normal Krebs solution, after which the strain was returned to 0%. Following a 60–90‐min rest, the strip was exposed to the drug, and stress–strain measurements were repeated. In the absence of any drug, a rest period of 60 min was generally sufficient for the stress–strain relationship to return to its initial state. Data points corresponding to each strain under the same conditions were first averaged for strips from the same animal, and then the mean values for each animal were averaged across different animals. On each plot, the stress data for a given strain are shown as mean ± SD, with “*n*” indicating the total number of animals studied. The exact “*n*” for each group is specified in the figure legends. A statistical comparison of the stress data for each axial strain applied to the DSM strip was performed using a two‐way ANOVA test, where one factor was the presence of mucosa and the other, the presence of a drug. Pairwise differences were assessed using Tukey's Honestly Significant Difference (HSD) post hoc test. In each graph, significant differences in means (*p* < 0.05) are marked with an asterisk. Data analysis was conducted using Origin 8.5 software (OriginLab Corp.). To directly assess the magnitude of the change in stress under the influence of the drug, for each strain, the mean stress values in the presence of the drug were divided by the mean stress values in the absence of the drug (i.e., the controls). Qualitatively similar results were obtained by calculating the mean ± SD of the division of variable X (i.e., the stress values at a given strain in the presence of the drug) by variable Y (i.e., the control stress values at the same strain in the absence of the drug), which are presented in Figure S1.

## 3 Results

### 3.1 Stretch‐Dependent Properties of Bladder Strips Under Control Conditions

**FIGURE 1:**
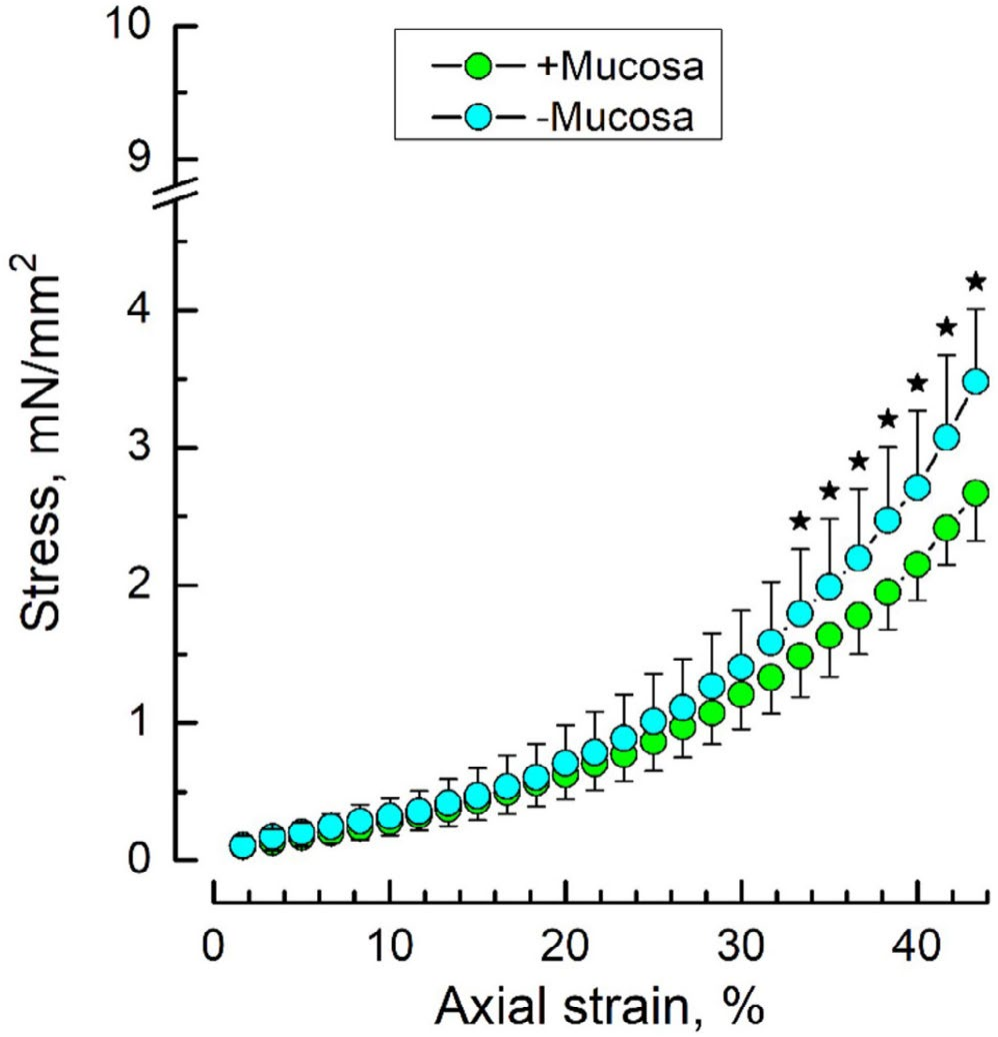
The presence of mucosa reduces the stress–strain dependency in rat bladder DSM strips. The figure shows stress–strain dependencies for mucosa‐intact (green) and mucosa‐devoid (light blue) rat DSM strips; data points are presented as mean ± SD, *n* = 12 for each condition; star marks indicate significantly different data points (*p* < 0.05) between mucosa‐intact and mucosa‐devoid preparations, as determined by Tukey's HSD test.

Under drug‐free conditions, stress–strain dependencies of DSM strips with and without urothelium (i.e., mucosa‐intact and mucosa‐devoid, respectively) were related to each other exactly as was described in our previous study [14], namely, in the whole range of applied strains, the stress developed by the DSM strip was consistently higher in the absence of mucosa as compared to its presence (Figure 1). Moreover, such a relationship between the stress–strain dependencies for mucosa‐intact and mucosa‐devoid DSM strips was characteristic of all animals, which allowed all individual dependencies acquired under drug‐free conditions to be pooled for statistical purposes to generate the common control means with which drug‐affected dependencies were compared. Higher stress developed by mucosa‐devoid DSM strips suggests that under normal conditions, upon stretching, the urothelium releases mediator(s) that produce a cumulative relaxing effect on DSM.

### 3.2 TREK1

**FIGURE 2:**
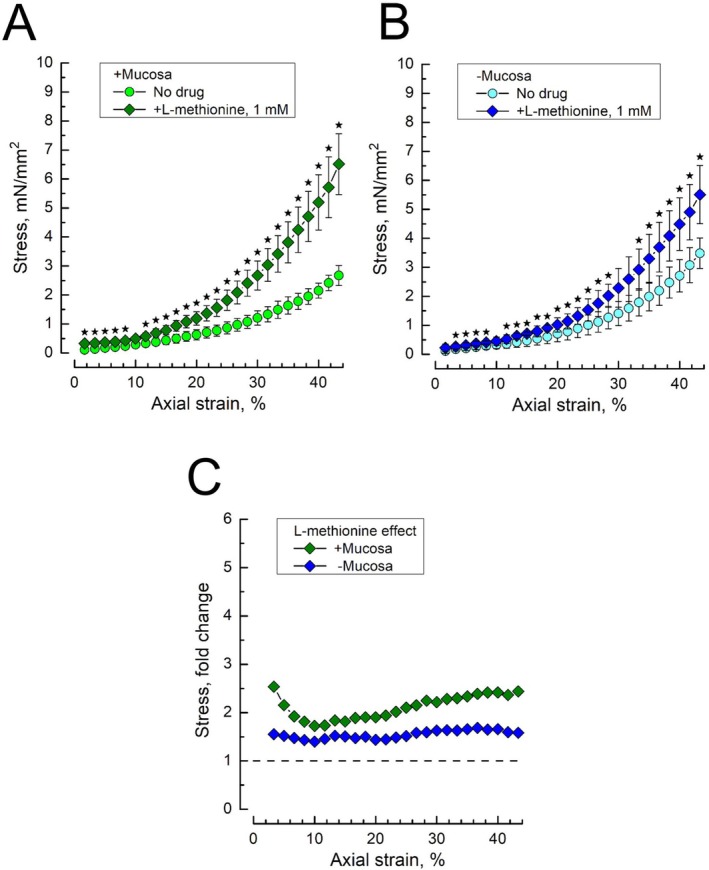
The inhibitor of K^+^‐selective TREK1 channel, L‐methionine, enhances stress–strain dependencies in both mucosa‐intact and mucosa‐devoid rat bladder DSM strips. (A, B) Stress–strain dependencies of mucosa‐intact (A) and mucosa‐devoid (B) DSM strips under no drug (control) conditions (circles) and in the presence of L‐methionine (1 mM, diamonds); data points—mean ± SD; no‐drug *n* = 12, L‐methionine *n* = 7; star marks indicate significantly different data points (*p* < 0.05) between drug and no drug, as determined by Tukey's HSD test. (C) The ratios of the L‐methionine‐affected to control stress–strain dependencies for the mucosa‐intact (+Mucosa, green diamonds) and mucosa‐devoid (−Mucosa, blue diamonds) DSM strips, obtained by dividing the mean stress values under the influence of the drug by the mean stress values in the absence of the drug (i.e., the controls); see also Figure S1A which presents the calculation of the mean ± SD of the division of variable X (i.e., the stress values at a given strain in the presence of the drug) by variable Y (i.e., the control stress values at the same strain in the absence of the drug).

The involvement of the 2P‐domain K^+^ channel, TREK1, in the stretch response of rat bladder DSM strips was assessed with its established inhibitor, L‐methionine, at 1 mM, a standard concentration used in similar studies (e.g., [13]). The effects of L‐methionine (1 mM) on DSM stress–strain dependencies are presented in Figure 2A–C. In DSM strips with intact mucosa, L‐methionine (1 mM) caused an increase in stress in the whole range of applied strains (Figure 2A,C), which is generally consistent with the previously drawn conclusion that TREK1 is responsible for the stretch‐dependent DSM relaxation [13, 18]. On average, L‐methionine‐stimulated stress increase in mucosa‐intact DSM strips constituted around 2.1‐fold, with a minimum of 1.7‐fold at strains around 10% (Figure 2C). It should be noted, however, that at strains below 10%, due to rather small and close stress values under control conditions and the drug action, calculation of their ratio results in considerable uncertainty, which is why it should be interpreted with caution.

In mucosa‐devoid DSM strips, L‐methionine also produced enhancement of stress, which, however, was smaller in size (~1.5‐fold) and less strain‐dependent as compared to the mucosa‐intact ones (Figure 2B,C). These results confirm that TREK1 function exerts relaxing action on DSM by being directly expressed in the DSM cells, as was postulated before [13], however, the presence of urothelium facilitates TREK1‐mediated relaxing action most likely due to involvement of urothelial TREK1 in the control of the release of some urothelial relaxing factor.

### 3.3 TRPV4

Expression and function of mechanosensitive cationic, Ca^2+^‐permeable channel, TRPV4, were demonstrated in both urothelium and DSM [7, 8, 9, 15, 21]. To examine the involvement of TRPV4 in the response of rat bladder DSM strips to stretching, the selective TRPV4 agonist, GSK1016790A, and the antagonist, HC067047, were used. For both GSK1016790A and HC067047, a fairly broad range of effective concentrations is reported in the literature: from ~0.1 μM for the effects on TRPV4‐mediated membrane current or intracellular calcium rise in isolated cells to 10 μM for bladder instillation studies [7, 15, 16, 21, 22, 23]. Importantly, both drugs proved ineffective in bladders from *Trpv4*‐knockout mice, suggesting a lack of off‐target actions [15, 16]. Given the need to achieve a near‐saturating effect on TRPV4 channel under limited diffusion in the multicellular bladder preparations, 1 μM of GSK1016790A and 10 μM HC067047 were selected for our experiments.

**FIGURE 3:**
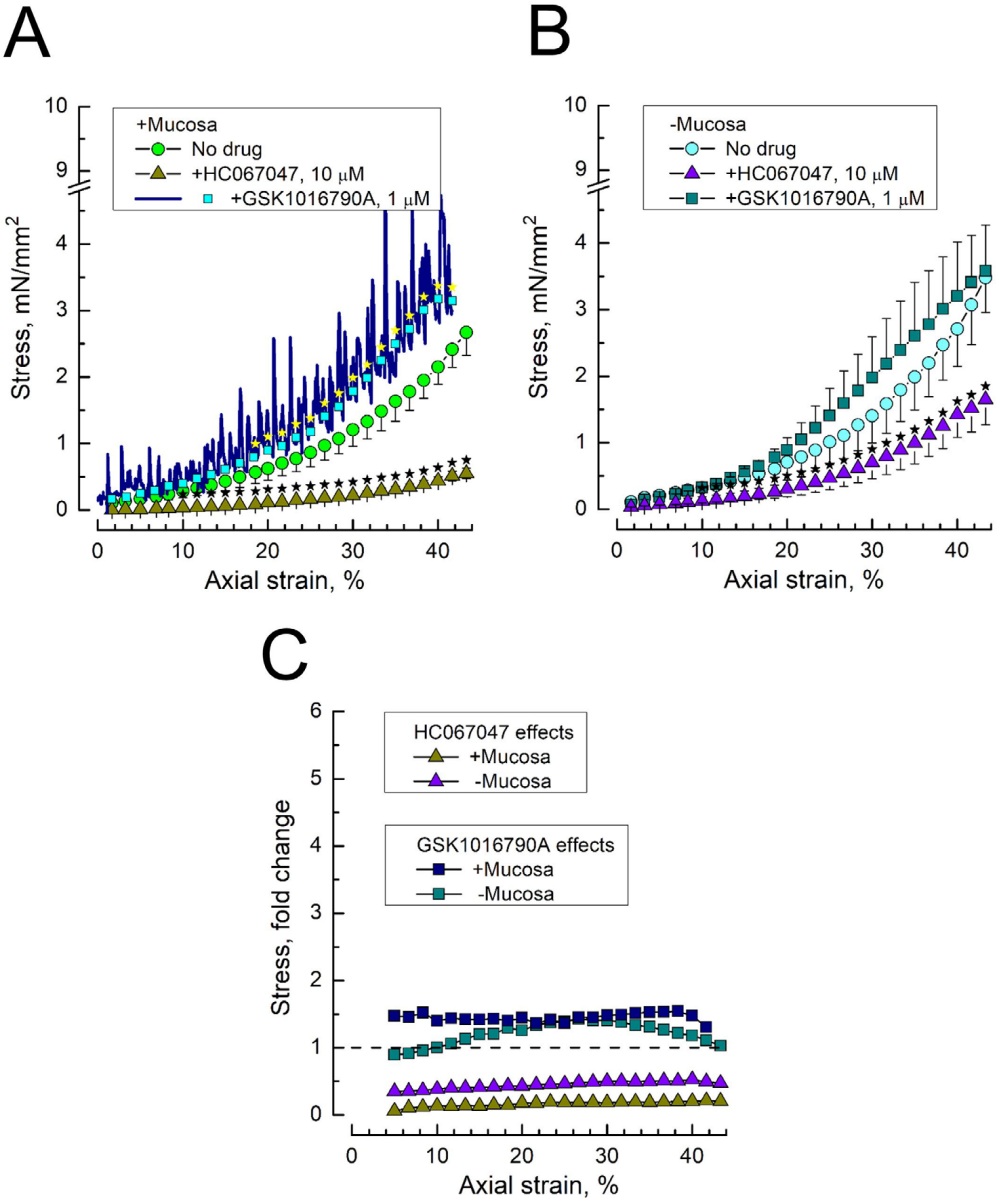
TRPV4 channel inhibitor, HC067047, suppresses, and TRPV4 channel activator, GSK1016790A, augments stress–strain dependencies in mucosa‐intact and mucosa‐devoid rat bladder DSM strips. (A, B) Stress–strain dependencies of mucosa‐intact (A) and mucosa‐devoid (B) DSM strips under no drug (control) conditions (circles) and in the presence of HC067047 (10 μM, triangles) or GSK1016790A (1 μM, squares); data points—mean ± SD; no drug *n* = 12, each drug *n* = 5; star marks indicate significantly different data points (*p* < 0.05) between drug and no drug, as determined by Tukey's HSD test; small cyan squares superimposed on continuous navy curve in A represent data interpolation for the used discrete values of strain (see text for details). (C) The ratios of the stress–strain dependency in the presence of HC067047 (triangles) or GSK1016790A (squares) to the control one for the mucosa‐intact (+Mucosa, dark yellow and navy) and mucosa‐devoid (−Mucosa, violet and dark cyan) DSM strips, obtained by dividing the mean stress values under the influence of the drug by the mean stress values in the absence of the drug (i.e., the controls); see also Figure S1A which presents the calculation of the mean ± SD of the division of variable X (i.e., the stress values at a given strain in the presence of the drug) by variable Y (i.e., the control stress values at the same strain in the absence of the drug).

TRPV4 antagonist HC067047 (10 μM) suppressed stress–strain dependencies in both mucosa‐intact (Figure 3A) and mucosa‐devoid DSM strips (Figure 3B). However, the residual level of stress in the presence of HC067047 in the mucosa‐intact preparations was notably lower as compared to the mucosa‐devoid ones (Figure 3A,B, triangles). These data are consistent with TRPV4 expression and function in DSM, whose stretching causes the TRPV4‐dependent increase of DSM tone, while TRPV4 blockade by HC067047 prevents such an increase. The fact that in the presence of urothelium, blockade of TRPV4 by HC067047 leads to even stronger reduction of stress is indicative of the TRPV4‐dependent release by stretched urothelium of some additional pro‐contractile factor.

As expected for the cationic, Ca^2+^‐permeable channel, the use of TRPV4 agonist GSK1016790A (1 μM) increased stress (Figure 3A,B). The GSK1016790A‐induced stress increase was not statistically significant in both mucosa‐free and mucosa‐intact preparations, with the only notable difference being a considerable enhancement of spontaneous contractile activity in mucosa‐intact DSM (Figure 3A, navy curve). An increase in spontaneous activity prevented the identification of a relaxation plateau phase during a stepwise increase in strain, forcing us to present the stress–strain relationship for mucosa‐intact strips in the presence of GSK1016790A as a continuous average curve of respective original recordings (Figure 3A, navy curve). To bring this curve to the format valid for comparison with other stress–strain dependencies, its values at applied discrete strains were fitted with an exponential growth function, and the values of the function at those strains (Figure 3A, cyan squares) were used in further analysis. The small size of the GSK1016790A‐induced increase of stress and little difference in its maximal level in the mucosa‐intact and mucosa‐devoid DSM strips suggested to us that stretch per se leads to nearly full engagement of TRPV4 in the DSM cells, and the use of its chemical activator cannot produce much more of an additional effect.

### 3.4 PIEZO1

Expression of mechanoactivated PIEZO1 and its function in stretch‐evoked ATP release has been mostly demonstrated for urothelial cells [11], although it may also be present in other layers of the bladder wall (lamina propria, detrusor) [10] where its function is not completely understood. As a tool to influence PIEZO1 in our stress–strain measurements we used PIEZO1 chemical activator, Yoda1. In the original study with Yoda1, a poor drug solubility was observed above 10 μM even with DMSO as a vehicle, leading to uncertainty regarding the actual value of its EC_50_ [17]. To avoid insolubility issues, 10 μM of Yoda1 was used in our experiments.

**FIGURE 4:**
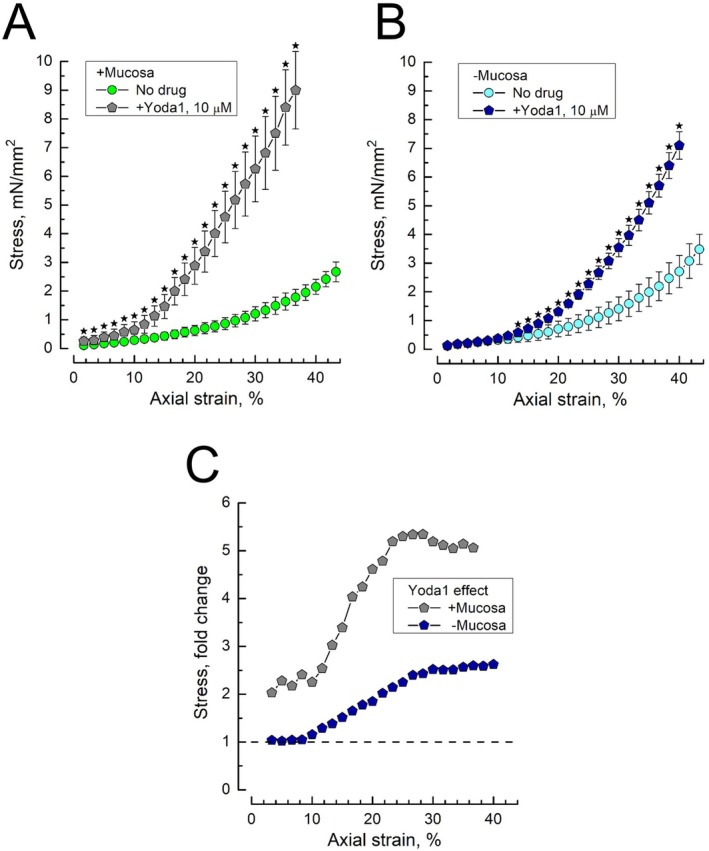
Chemical activator of PIEZO1 channel, Yoda1, enhances stress–strain dependencies in both mucosa‐intact and mucosa‐devoid rat bladder DSM strips. (A, B) Stress–strain dependencies of mucosa‐intact (A) and mucosa‐devoid (B) DSM strips under no drug (control) conditions (circles) and in the presence of Yoda1 (10 μM, pentagons); data points—mean ± SD; no drug *n* = 12, drug *n* = 9; star marks indicate significantly different data points (*p* < 0.05) between drug and no drug, as determined by Tukey's HSD test. (C) The ratios of the Yoda1‐affected to control stress–strain dependencies for the mucosa‐intact (+Mucosa, gray pentagons) and mucosa‐devoid (−Mucosa, navy pentagons) DSM strips, obtained by dividing the mean stress values under the influence of the drug by the mean stress values in the absence of the drug (i.e., the controls); see also Figure S1A which presents the calculation of the mean ± SD of the division of variable X (i.e., the stress values at a given strain in the presence of the drug) by variable Y (i.e., the control stress values at the same strain in the absence of the drug).

As shown in Figure 4A,B, Yoda1 (10 μM) markedly enhanced stress in both mucosa‐intact and mucosa‐devoid DSM strips. In mucosa‐intact DSM strips (Figure 4A), the enhancement was stronger and revealed quite complex strain‐dependency as compared to the Yoda1‐free condition: at low strains of up to 7% it was quite modest (around 2‐fold) after which it progressively increased to about 5.3‐fold at a strain of 25% and then even slightly decreased at higher strains (Figure 4C). This suggests that the role of the PIEZO1 channel in determining the mechanical properties of the bladder wall depends on the magnitude of the mechanical stimulus.

Removal of mucosa (Figure 4B) essentially reduced the ability of Yoda1 to enhance stress; however, the general strain‐dependency remained quite similar: almost no increase of stress at strains below 7%, its progressive increase up to about 2.5‐fold around 25% strain, and saturation afterwards (Figure 4C). Thus, PIEZO1 is functionally present in the DSM cells, but the full‐fledged reaction of the bladder wall to the stretch requires activation of the urothelial PIEZO1 as well.

Unfortunately, pharmacological tools specific to PIEZO2 are not available yet [24], which made it impossible to assess PIEZO2's involvement using a pharmacological approach.

### 3.5 Stress–Strain Dependencies Are Determined by Active Mechanosensation

**FIGURE 5:**
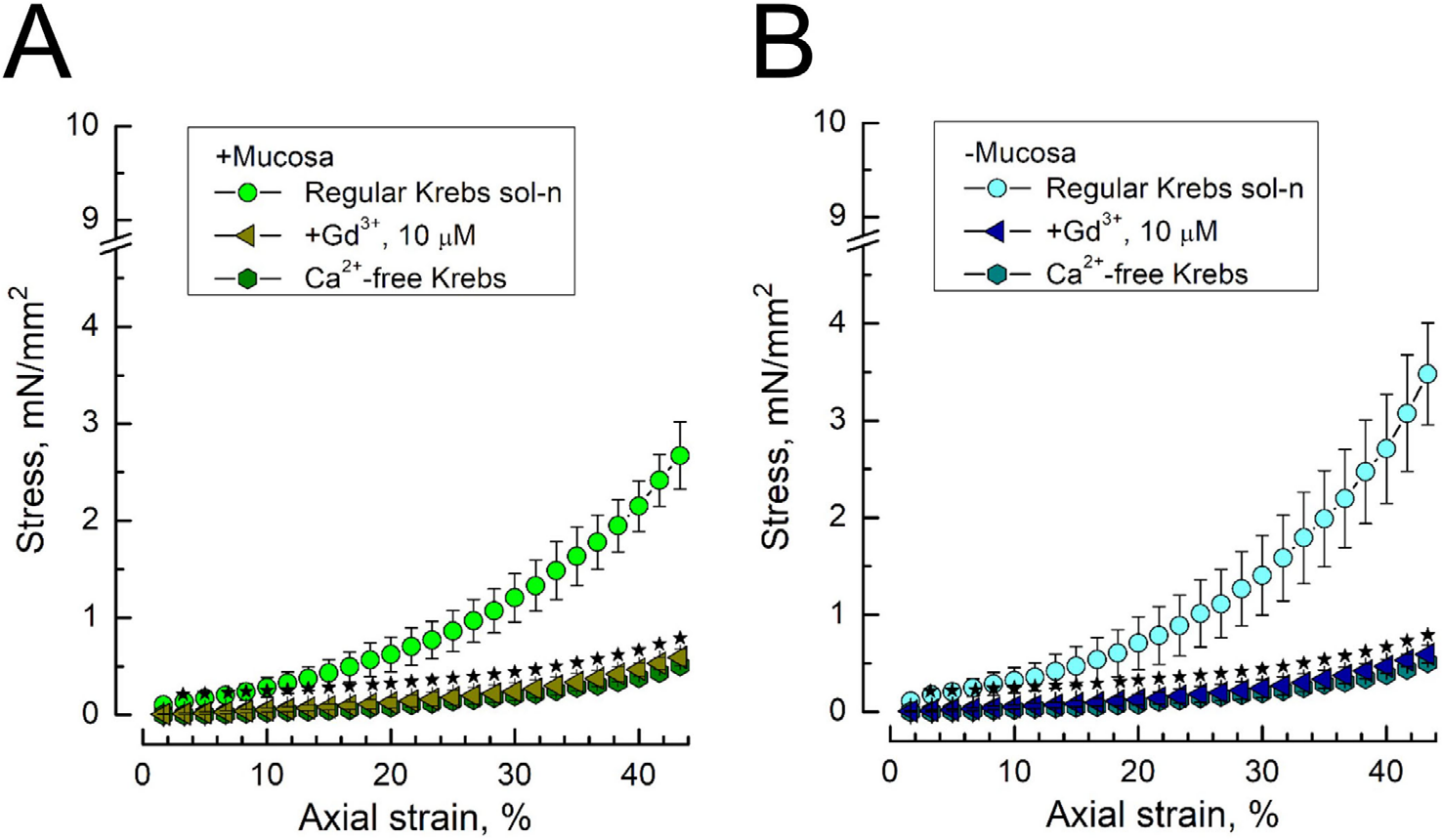
Stress–strain dependencies in both mucosa‐intact and mucosa‐devoid rat bladder DSM strips are suppressed by a non‐specific inhibitor of mechanogated ion channels, gadolinium, and by Ca^2+^‐free external medium. (A, B) Stress–strain dependencies of mucosa‐intact (A) and mucosa‐devoid (B) DSM strips under control conditions (circles, regular Krebs sol‐n) and in the presence of Gd^3+^ (10 μM, triangles) or nominally Ca^2+^‐free Krebs (hexagons); data points—mean ± SD; control *n* = 12, Gd^3+^ and Ca^2+^‐free *n* = 5 each; star marks indicate significantly different data points (*p* < 0.05) between regular Krebs and Ca^2+^‐free or Gd^3+^ conditions, as determined by Tukey's HSD test.

To check to what extent elastic properties of bladder tissues per se contribute to the observed stress–strain dependencies, we have performed their measurements in the presence of a non‐specific inhibitor of stretch‐activated ion channels, gadolinium [25], and in nominally Ca^2+^‐free extracellular Krebs solution to prevent Ca^2+^ entry. Both the Ca^2+^‐free Krebs solution as well as the addition of Gd^3+^ (10 μM) to the regular one almost completely suppressed any development of stress in response to the application of strain, irrespective of the presence of mucosa (Figure 5). This suggests that, at least in the range of applied strains, the developed stress is determined by the concerted action of mechanosensitive channels linked to Ca^2+^ influx, and not by the passive elastic properties of the bladder tissue.

## 4 Discussion

In this article, we report on three major findings: (1) the response to stretch of the rat bladder wall ex vivo is determined by the mechanisms of active mechanosensation rather than by the elastic properties of the bladder wall tissue; (2) upon stretch, the urothelium releases chemical mediator(s) that exert a cumulative relaxing effect on DSM; (3) the urothelial layer and DSM layer are both endowed with active mechanosensory mechanisms relying on the functioning of mechanoactivated PIEZO1, TRPV4, and TREK1 channels. These findings were obtained by applying a pharmacological approach in which we asked how activation or inhibition of a certain channel by specific pharmacological tools could influence the DSM strip's response to stretching. To answer this question, we used concentrations of the drugs which according to the literature produce near‐saturating effects on the channel, and the absence of the full concentration‐response characterization of each drug's action may be considered a limitation of this study. Below, we discuss the possible roles of each of the studied channel.

### 4.1 TREK1

**FIGURE 6:**
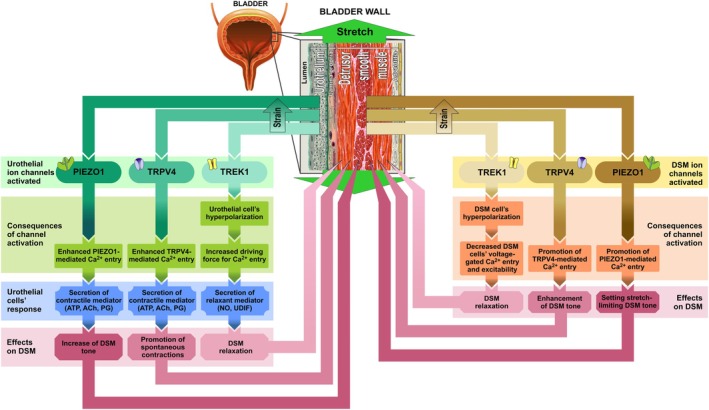
Proposed involvement of mechanosensitive ion channels, K^+^‐selective TREK1 and Ca^2+^‐permeable TRPV4 and PIEZO1, in regulating decentralized ex vivo rat bladder wall local contractility in response to stretch. The top part of the figure shows the layers of the bladder wall, the left section refers to ion channels expressed in urothelial cells, and the right section refers to ion channels expressed in detrusor smooth muscle (DSM) cells.

The fact that TREK1 inhibition by L‐methionine in mucosa‐devoid DSM strips caused an increase of stretch‐evoked stress well agrees with the relaxant function of this K^+^‐selective channel in DSM [13, 18]. It is thought that activation of TREK1 in response to stretch produces DSM cells' hyperpolarization, followed by the reduction of their excitability and voltage‐gated calcium entry during the bladder filling phase (Figure 6, right section, left‐hand blocks) allowing it to accommodate an increasing volume of the urine [13, 18]. Thus, it is natural to expect that elimination of stretch‐evoked, TREK1‐mediated hyperpolarization by L‐methionine would result in higher mucosa‐devoid DSM strips' stress at a given strain, which was observed in our experiments (Figure 2A).

What is more intriguing is that preserving intact mucosa further enhances L‐methionine‐evoked stress, indicating that in the presence of urothelium, the stretch‐induced, TREK1‐mediated relaxant effect on the DSM becomes stronger. This can only happen if the activation of urothelial TREK1 is linked to the release of mediator(s) that exert DSM relaxation. Urothelial cells are non‐excitable, meaning that their Ca^2+^ entry is supported by the functioning of cationic, Ca^2+^‐permeable non‐voltage‐gated channels. TREK1‐mediated hyperpolarization of urothelial cells would enhance Ca^2+^ entry through such channels by increasing the driving force for Ca^2+^ entry, and promote secretion (Figure 6, left section, right‐hand blocks). The bladder urothelium releases several mediators, including acetylcholine (ACh), ATP, nitric oxide (NO), prostaglandins, neuropeptides, and some enigmatic urothelium‐derived inhibitory factor (UDIF) that modulate DSM contraction and relaxation via complex autocrine and paracrine interactions [4, 5, 26, 27, 28]. Moreover, these mediators can be released from the basolateral surface of urothelial cells and diffuse into deeper tissue layers, thus transmitting the information about the state of the mucosa to underlying nerve processes, myofibroblasts, interstitial cells, and musculature (e.g., [29]). Excision of bladder wall strips does not disrupt this communication, as demonstrated in studies on mucosa‐intact and mucosa‐devoid preparations reported in the literature (e.g., [30]).

Among the mediators secreted by the urothelium, ATP, NO, and ACh are known to be specifically released in response to mechanical stimuli [5, 28, 31]. Furthermore, by acting in an autocrine/paracrine manner, ACh has been suggested to promote urothelial release of ATP, UDIF, and NO [32]. Although NO per se is unlikely to act as a direct relaxant for the DSM [5, 32, 33], it may influence muscle tone indirectly by modulating interstitial cells in the suburothelium [5]. The role of UDIF in this process cannot be excluded either.

Furthermore, ATP in the bladder is known to elicit a dual response: a contraction followed by a sustained relaxation (e.g., [34]). Contractions are mediated by ATP binding mainly to ionotropic P2X1 purinoreceptors on DSM cells [33, 34], whereas ATP‐activated DSM cells' metabotropic P2Y purinoreceptors and P1 purinoreceptors activated by ATP metabolite, adenosine, are thought to be responsible for relaxation [34]. It is feasible to suggest that ATP of neuronal origin, which is co‐released with ACh from intramural efferent nerve endings during excitation, induces DSM contractions. On the other hand, ATP released from urothelium in response to stretching may not only excite bladder afferent nerves but also promote DSM relaxation. In the latter case, the urothelial TREK1 channel may be postulated to play an important role in regulating ATP release from urothelium that is specifically targeted to DSM cells.

Thus, based on our data, one can conclude that the mechanosensitive TREK1 channel is coupled with bladder wall relaxation via two mechanisms: (1) decreasing excitability of DSM per se and (2) release of relaxant mediator(s) from the urothelium.

### 4.2 TRPV4

Our data on mucosa‐intact DSM strips indicate that TRPV4 is present in DSM cells, where its functioning as a stretch‐activated Ca^2+^‐permeable channel contributes to the stress in response to the application of strain (Figure 6, right section, middle blocks). Consequently, blockade of TRPV4 in DSM cells by CH067047 leads to the stress decrease. The fact that with intact mucosa, HC067047 produced a stronger decrease of stress suggests that TRPV4 is also present in urothelial cells and that its stretch‐dependent activation in these cells is coupled to the release of the mediator(s) with contractile action on DSM (Figure 6, left section, middle blocks). The candidates for such mediators include: ATP, ACh, prostaglandins [5, 35].

TRPV4 functioning as a stretch‐activated Ca^2+^‐permeable channel in both DSM and urothelial cells was also supported by the action of TRPV4 agonist GSK1016790A. Irrespective of the presence of mucosa, GSK1016790A was able to enhance DSM strips' stress only marginally, suggesting that stretch per se is sufficient to nearly fully activate TRPV4, leaving little room for its further activation by chemical agonist. However, in mucosa‐intact but not mucosa‐devoid DSM strips, GSK1016790A strongly promoted spontaneous contractility, which agrees well with our previous observations [36]. The role of urothelial TRPV4 in supporting spontaneous contractions was convincingly demonstrated in TRPV4‐knockout mice whose bladder strips were characterized by a significant reduction of the amplitude but not duration and frequency of spontaneous contractions compared to the wild‐type ones and by their nearly complete cessation in urothelium‐free strips from both groups of animals [37]. Spontaneous contractile activity of DSM works against the bladder compliance in the filling phase to allow, as thought, the individual muscle bundles to adjust length in response to filling [38]. Thus, our data demonstrate a role for TRPV4 in urothelium‐mediated conversion of mechanical stretch into non‐voiding spontaneous autonomous DSM contractions (Figure 6, left section, middle blocks), although the contribution of urothelial TRPV4 to previously postulated stretch‐evoked ATP release [8, 21, 39] cannot be excluded either.

### 4.3 PIEZO1

The presence of the PIEZO1 channel was documented in virtually all cell types of the bladder of several species: in the detrusor layer and the urothelium of rats [40], in the urothelium, the interstitial cells, and detrusor of mice [10, 11, 41], in the urothelium of human bladder [11, 42]. Most data point to the urothelial localization and function of PIEZO1. However, the precise expression patterns as well as the consequences of its activation seem to be species‐specific. In mouse urothelial cells, mechanical stretch stimuli provoked a PIEZO1‐dependent increase in cytosolic Ca^2+^ concentrations ([Ca^2+^]_i_), leading to potent ATP release [11]. In human bladder strips with intact urothelium, activation of PIEZO1 by Yoda1 was found to enhance spontaneous contractions and to induce the release of ATP and ACh from cultured human urothelial cells [42].

Given a considerable overlap in the urothelial PIEZO1 and TRPV4 expression and function in ATP release from urothelium, based on the studies in the mouse bladder, it was suggested that these channels are different in terms of the intensity of mechanical force that they sense, with PIEZO1 acting as a more sensitive mechanoreceptor [11]. Moreover, it was also proposed that PIEZO1 and TRPV4 may be coupled to different ATP release mechanisms, conductive one via engaging the plasma membrane ATP‐permeable pores (like anion channels or connexin hemichannels) and exocytotic one [11, 21].

Our data on the measurements of stress–strain dependencies of mucosa‐intact and mucosa‐devoid rat DSM strips and their sensitivity to Yoda1 are consistent with the presence of PIEZO1 in both urothelium and DSM (Figure 6, leftmost and rightmost blocks). In mucosa‐devoid DSM strips, Yoda1 starts to progressively contribute to the development of stress at strains above 10%, and its effect saturates above 30% (Figure 4C). Inconstancy of Yoda1 effects in the whole range of applied strains indicates that in DSM, the efficiency of the PIEZO1 chemical activator depends on the size of the mechanical stimulus.

In the presence of mucosa, the effects of Yoda1 strongly increased, but did not result in the appearance of spontaneous DSM contractions. This contrasts with what has been reported for human bladder strips [42] and with what we observed for the TRPV4 chemical agonist, GSK1016790A (Figure 3A). In the presence of mucosa, Yoda1 enhanced stress even at strains below 10%, suggesting that it can increase basal activity of urothelial cells‐localized PIEZO1 with concomitant Ca^2+^ entry, promoting the secretion of the mediator with contractile action of DSM, most likely ACh or ATP (Figure 6, left section, left‐hand blocks). Thus, in rat bladder urothelial cells and DSM cells, PIEZO1 concurrently acts as a stretch sensor to increase bladder wall tone with increasing stretch.

## 5 Conclusions and Implications

Our data show that all three mechanosensory channels, TREK1, TRPV4, and PIEZO1, are functionally present in the rat bladder DSM and urothelium to provide for the local stretch‐dependent regulation of bladder wall contraction/relaxation independent of the nervous system. Of these channels, the activation of DSM cells‐localized K^+^‐selective TREK1 promotes DSM relaxation via hyperpolarization of DSM cells and the decrease of their voltage‐gated Ca^2+^ entry (Figure 6, right section, left‐hand blocks). Activation of TREK1 expressed in urothelial cells further contributes to the DSM relaxation via the release from urothelium of the mediator with the relaxant action on DSM. The likely candidates for these mediators are NO, ATP, and UDIF (Figure 6, left section, right‐hand blocks).

Nearly full engagement in response to the stretching of the non‐voltage‐gated, Ca^2+^‐permeable TRPV4 channel, functionally expressed in DSM cells, is indicative of its low threshold of activation by mechanical stimuli. Due to this, TRPV4 may contribute to the gradual increase of DSM tone during TREK1‐dependent hyperpolarization that accompanies bladder filling (Figure 6, right section, middle blocks). Generation of spontaneous contractions in response to the activation of the urothelial cells‐localized TRPV4 well agrees with the release of the excitatory mediator(s), which exert little effect on DSM tone, but by acting on DSM cells, and may be the bladder interstitial cells, evoke the generation of spontaneous contractions (Figure 6, left section, middle blocks). These mediators may include ATP, ACh, and prostaglandins [5, 35].

Activation of the Ca^2+^‐permeable PIEZO1 channel exerts a much more potent effect on the development of DSM tone in response to stretching as compared to TRPV4. In DSM cells, PIEZO1 exhibits a higher threshold but steeper sensitivity of activation by stretching than TRPV4. Activation of urothelial PIEZO1 leads to the release of the mediators with contractile action on DSM, like ACh or ATP. Overall, PIEZO1 activation may play a role in limiting the extent of bladder distention during filling (Figure 6, leftmost and rightmost blocks).

The specificity of the coupling between a certain type of mechanosensitive ion channel and the release of a certain mediator by urothelium may be supported by the specialized urothelial cell populations. The possibility of such specialization was recently demonstrated for DSM cells [43]. Formation of the microdomains in urothelial cells with preferred communication between the channel and the mediator's secretion machinery is possible as well. Finally, the possibility for the same urothelial mediator (e.g., ATP) to produce different effects on DSM cells may be determined by spatial relationships between the site of release of the mediator and the target of its action. Answering these questions requires future studies.

In summary, the effects discovered have the following significance for bladder physiology. The function of TREK1 in both the DSM and urothelium induces relaxation of the bladder wall, thus allowing an expansion of bladder volume without a significant increase in intravesical pressure during filling. Simultaneously, TRPV4 activity ensures a gradual increase in bladder wall stiffness with increasing volume. PIEZO1, as exhibiting a higher threshold and steeper sensitivity of activation by stretching than TRPV4, intervenes at later stages to ultimately limit the increase in bladder volume. As suggested before, spontaneous contractile activity serves to adjust the length of the individual muscle bundles in response to filling [38]. Based on our current and previous studies [36], as well as those of others (e.g., [37]), spontaneous contractile activity appears to be dependent on TRPV4. Thus, it can be suggested that, in addition to ensuring a gradual increase in bladder wall stiffness with increasing volume, TRPV4 contributes to the adjustment of muscle bundles to variations in bladder volume.

In theory, in experiments on isolated bladder strips, transmitters released by the urothelium can reach underlying tissues and influence contractility not locally by crossing lamina propria but through the surrounding bath solution. Furthermore, this experimental design has its limitations in that it does not distinguish between apical and basolateral release of the transmitters. Thus, the physiological relevance of the results obtained with its help, which indicate that active mechanosensitivity involves the release of urothelial factors that act on the DSM, could be questioned. However, our recent comparative study of the stress‐volume relationship of the whole bladders ex vivo and the stress–strain relationship of mucosa‐intact bladder strips from normal rats and rats with modeled type 2 diabetes yielded similar results [14]. This indicates that the main pathways in which urothelially‐released factors affect contractility in isolated bladder strips remain the same as in the intact bladder and are therefore physiologically relevant, while the action through the surrounding bath solution, if any, is negligible.

## Author Contributions

Igor B. Philyppov, Ganna V. Sotkis, Semen Yelyashov, and Valeri G. Naidenov performed tensiometric experiments and results analysis; Igor B. Philyppov and Yaroslav M. Shuba initiated the project and designed the study; Yaroslav M. Shuba created the figures and wrote the draft of the paper; all authors read and approved the manuscript.

## Funding

This work was supported by the National Research Foundation of Ukraine grant 2020.02/0189.

## Conflicts of Interest

The authors declare no conflicts of interest.

## Supporting information


**Figure S1:** Evaluation of the magnitude of the effect of drugs, specific for mechanosensitive ion channels, on the stress–strain relationship of rat bladder DSM strips. A–D: Results of the calculation the mean ± SD of the division of variable X (i.e., the stress values at a given strain in the presence of the drug) by variable Y (i.e., the control stress values at the same strain in the absence of the drug) for the TREK1 channel inhibitor, L‐methionine (A), the TRPV4 channel agonist, GSK1016790A (B), the TRPV4 channel inhibitor, HC‐067047 (C), and the PIEZO1 channel activator, Yoda1 (D), according to the formulas [Ruiz Espejo Mariano, 2015]:
The data used to construct plots A–D are those presented in panels A, B of Figure 2, Figure 3 and Figure 4 of the main text.

## Data Availability

The data that support the findings of this study are available from the corresponding author upon reasonable request.

## References

[1] I. Milsom and M. Gyhagen , “The Prevalence of Urinary Incontinence,” Climacteric 22, no. 3 (2019): 217–222. 30572737 10.1080/13697137.2018.1543263

[2] W. C. de Groat , D. Griffiths , and N. Yoshimura , “Neural Control of the Lower Urinary Tract,” Comprehensive Physiology 5, no. 1 (2015): 327–396. 25589273 10.1002/cphy.c130056 PMC4480926

[3] S. S. Ranade , R. Syeda , and A. Patapoutian , “Mechanically Activated Ion Channels,” Neuron 87, no. 6 (2015): 1162–1179. 26402601 10.1016/j.neuron.2015.08.032 PMC4582600

[4] L. A. Birder , M. Ruggieri , M. Takeda , et al., “How Does the Urothelium Affect Bladder Function in Health and Disease? ICI‐RS 2011,” Neurourology and Urodynamics 31, no. 3 (2012): 293–299. 22275289 10.1002/nau.22195 PMC3309105

[5] D. Sellers , R. Chess‐Williams , and M. C. Michel , “Modulation of Lower Urinary Tract Smooth Muscle Contraction and Relaxation by the Urothelium,” Naunyn‐Schmiedeberg's Archives of Pharmacology 391, no. 7 (2018): 675–694. 29808232 10.1007/s00210-018-1510-8

[6] I. Araki , S. Du , H. Kobayashi , et al., “Roles of Mechanosensitive Ion Channels in Bladder Sensory Transduction and Overactive Bladder,” International Journal of Urology 15, no. 8 (2008): 681–687. 18462357 10.1111/j.1442-2042.2008.02052.x

[7] A. Isogai , K. Lee , R. Mitsui , and H. Hashitani , “Functional Coupling of TRPV4 Channels and BK Channels in Regulating Spontaneous Contractions of the Guinea Pig Urinary Bladder,” Pflügers Archiv 468, no. 9 (2016): 1573–1585. 27497848 10.1007/s00424-016-1863-0

[8] T. Mochizuki , T. Sokabe , I. Araki , et al., “The TRPV4 Cation Channel Mediates Stretch‐Evoked Ca2+ Influx and ATP Release in Primary Urothelial Cell Cultures,” Journal of Biological Chemistry 284, no. 32 (2009): 21257–21264. 19531473 10.1074/jbc.M109.020206 PMC2755849

[9] Y. Wu , J. Qi , C. Wu , and W. Rong , “Emerging Roles of the TRPV4 Channel in Bladder Physiology and Dysfunction,” Journal of Physiology 599, no. 1 (2021): 39–47. 33052604 10.1113/JP279776 PMC11933886

[10] M. G. Dalghi , W. G. Ruiz , D. R. Clayton , et al., “Functional Roles for PIEZO1 and PIEZO2 in Urothelial Mechanotransduction and Lower Urinary Tract Interoception,” JCI Insight 6, no. 19 (2021): e152984. 34464353 10.1172/jci.insight.152984 PMC8525643

[11] T. Miyamoto , T. Mochizuki , H. Nakagomi , et al., “Functional Role for Piezo1 in Stretch‐Evoked Ca2+ Influx and ATP Release in Urothelial Cell Cultures,” Journal of Biological Chemistry 289, no. 23 (2014): 16565–16575. 24759099 10.1074/jbc.M113.528638 PMC4047422

[12] K. L. Marshall , D. Saade , N. Ghitani , et al., “PIEZO2 in Sensory Neurons and Urothelial Cells Coordinates Urination,” Nature 588, no. 7837 (2020): 290–295. 33057202 10.1038/s41586-020-2830-7 PMC7725878

[13] Q. Lei , X. Q. Pan , S. Chang , S. B. Malkowicz , T. J. Guzzo , and A. P. Malykhina , “Response of the Human Detrusor to Stretch Is Regulated by TREK1, a Two‐Pore‐Domain (K2P) Mechano‐Gated Potassium Channel,” Journal of Physiology 592, no. 14 (2014): 3013–3030. 24801307 10.1113/jphysiol.2014.271718 PMC4214657

[14] I. B. Philyppov , G. V. Sotkis , A. O. Danshyna , S. I. Yelyashov , B. R. Sharopov , and Y. M. Shuba , “Impairment of Urinary Bladder Mechanical Properties in Rat Model of Type 2 Diabetes,” Neurourology and Urodynamics 41, no. 8 (2022): 1670–1678. 35979707 10.1002/nau.25024

[15] K. S. Thorneloe , A. C. Sulpizio , Z. Lin , et al., “N‐((1S)‐1‐{[4‐((2S)‐2‐{[(2,4‐Dichlorophenyl)sulfonyl]Amino}‐3‐Hydroxypropanoyl)‐1‐Piperazinyl]Carbonyl}‐3‐Methylbutyl)‐1‐Benzothiophene‐2‐Carboxamide (GSK1016790A), a Novel and Potent Transient Receptor Potential Vanilloid 4 Channel Agonist Induces Urinary Bladder Contraction and Hyperactivity: Part I,” Journal of Pharmacology and Experimental Therapeutics 326, no. 2 (2008): 432–442. 18499743 10.1124/jpet.108.139295

[16] W. Everaerts , X. Zhen , D. Ghosh , et al., “Inhibition of the Cation Channel TRPV4 Improves Bladder Function in Mice and Rats With Cyclophosphamide‐Induced Cystitis,” Proceedings of the National Academy of Sciences of the United States of America 107, no. 44 (2010): 19084–19089. 20956320 10.1073/pnas.1005333107 PMC2973867

[17] R. Syeda , J. Xu , A. E. Dubin , et al., “Chemical Activation of the Mechanotransduction Channel Piezo1,” eLife 4 (2015): e07369. 26001275 10.7554/eLife.07369 PMC4456433

[18] S. A. Baker , G. W. Hennig , J. Han , F. C. Britton , T. K. Smith , and S. D. Koh , “Methionine and Its Derivatives Increase Bladder Excitability by Inhibiting Stretch‐Dependent K(+) Channels,” British Journal of Pharmacology 153, no. 6 (2008): 1259–1271. 18204472 10.1038/sj.bjp.0707690 PMC2275456

[19] K. J. Park , S. A. Baker , S. Y. Cho , K. M. Sanders , and S. D. Koh , “Sulfur‐Containing Amino Acids Block Stretch‐Dependent K+ Channels and Nitrergic Responses in the Murine Colon,” British Journal of Pharmacology 144, no. 8 (2005): 1126–1137. 15700022 10.1038/sj.bjp.0706154 PMC1576098

[20] K. Braak and H. H. Frey , “Effects of Solvents and Detergents on the Contractions of Isolated Smooth Muscle Preparations,” Journal of Pharmacy and Pharmacology 42, no. 12 (1990): 837–841. 1983145 10.1111/j.2042-7158.1990.tb07036.x

[21] M. W. G. Roberts , G. Sui , R. Wu , et al., “TRPV4 Receptor as a Functional Sensory Molecule in Bladder Urothelium: Stretch‐Independent, Tissue‐Specific Actions and Pathological Implications,” FASEB Journal 34, no. 1 (2020): 263–286. 31914645 10.1096/fj.201900961RR PMC6973053

[22] Y. Deruyver , E. Weyne , K. Dewulf , et al., “Intravesical Activation of the Cation Channel TRPV4 Improves Bladder Function in a Rat Model for Detrusor Underactivity,” European Urology 74, no. 3 (2018): 336–345. 29875065 10.1016/j.eururo.2018.05.020

[23] B. M. Girard , S. E. Campbell , M. Perkins , et al., “TRPV4 Blockade Reduces Voiding Frequency, ATP Release, and Pelvic Sensitivity in Mice With Chronic Urothelial Overexpression of NGF,” American Journal of Physiology. Renal Physiology 317, no. 6 (2019): F1695–F1706. 31630542 10.1152/ajprenal.00147.2019 PMC6962511

[24] M. Szczot , A. R. Nickolls , R. M. Lam , and A. T. Chesler , “Form and Function of PIEZO2,” Annual Review of Biochemistry 90 (2021): 507–534. 10.1146/annurev-biochem-081720-023244 PMC8794004 34153212

[25] X. C. Yang and F. Sachs , “Block of Stretch‐Activated Ion Channels in Xenopus Oocytes by Gadolinium and Calcium Ions,” Science 243, no. 4894 Pt 1 (1989): 1068–1071. 2466333 10.1126/science.2466333

[26] N. N. Guan , L. E. Gustafsson , and K. Svennersten , “Inhibitory Effects of Urothelium‐Related Factors,” Basic & Clinical Pharmacology & Toxicology 121, no. 4 (2017): 220–224. 28371382 10.1111/bcpt.12785

[27] M. H. Hawthorn , C. R. Chapple , M. Cock , and R. Chess‐Williams , “Urothelium‐Derived Inhibitory Factor(s) Influences on Detrusor Muscle Contractility In Vitro,” British Journal of Pharmacology 129, no. 3 (2000): 416–419. 10711338 10.1038/sj.bjp.0703068 PMC1571854

[28] M. Winder , G. Tobin , D. Zupančič , and R. Romih , “Signalling Molecules in the Urothelium,” BioMed Research International 2014 (2014): 297295. 25177686 10.1155/2014/297295 PMC4142380

[29] P. Khandelwal , S. N. Abraham , and G. Apodaca , “Cell Biology and Physiology of the Uroepithelium,” American Journal of Physiology. Renal Physiology 297, no. 6 (2009): F1477–F1501. 19587142 10.1152/ajprenal.00327.2009 PMC2801337

[30] T. J. Heppner , H. C. Fallon , J. L. Rengo , et al., “Urothelium‐Derived Prostanoids Enhance Contractility of Urinary Bladder Smooth Muscle and Stimulate Bladder Afferent Nerve Activity in the Mouse,” American Journal of Physiology. Regulatory, Integrative and Comparative Physiology 327, no. 1 (2024): R97–R108. 38780425 10.1152/ajpregu.00084.2024 PMC11334713

[31] V. N. Mutafova‐Yambolieva , “Mechanosensitive Release of ATP in the Urinary Bladder Mucosa,” Purinergic Signal 21, no. 3 (2025): 413–428. 39541058 10.1007/s11302-024-10063-6 PMC12222603

[32] D. Giglio and G. Tobin , “Muscarinic Receptor Subtypes in the Lower Urinary Tract,” Pharmacology 83, no. 5 (2009): 259–269. 19295256 10.1159/000209255

[33] K. E. Andersson and A. Arner , “Urinary Bladder Contraction and Relaxation: Physiology and Pathophysiology,” Physiological Reviews 84, no. 3 (2004): 935–986. 15269341 10.1152/physrev.00038.2003

[34] P. Aronsson , M. Andersson , T. Ericsson , and D. Giglio , “Assessment and Characterization of Purinergic Contractions and Relaxations in the Rat Urinary Bladder,” Basic & Clinical Pharmacology & Toxicology 107, no. 1 (2010): 603–613. 20406212 10.1111/j.1742-7843.2010.00554.x

[35] I. Tanaka , K. Nagase , K. Tanase , Y. Aoki , H. Akino , and O. Yokoyama , “Modulation of Stretch Evoked Adenosine Triphosphate Release From Bladder Epithelium by Prostaglandin E_2_ ,” Journal of Urology 185, no. 1 (2011): 341–346. 21075387 10.1016/j.juro.2010.09.042

[36] I. B. Philyppov , G. V. Sotkis , A. Rock , et al., “Alterations in Detrusor Contractility in Rat Model of Bladder Cancer,” Scientific Reports 10, no. 1 (2020): 19651. 33184390 10.1038/s41598-020-76653-7 PMC7665011

[37] T. Gevaert , J. Vriens , A. Segal , et al., “Deletion of the Transient Receptor Potential Cation Channel TRPV4 Impairs Murine Bladder Voiding,” Journal of Clinical Investigation 117, no. 11 (2007): 3453–3462. 17948126 10.1172/JCI31766 PMC2030459

[38] A. F. Brading , “Spontaneous Activity of Lower Urinary Tract Smooth Muscles: Correlation Between Ion Channels and Tissue Function,” Journal of Physiology 570, no. Pt 1 (2006): 13–22. 16210349 10.1113/jphysiol.2005.097311 PMC1464291

[39] L. Birder , F. A. Kullmann , H. Lee , et al., “Activation of Urothelial Transient Receptor Potential Vanilloid 4 by 4alpha‐Phorbol 12,13‐Didecanoate Contributes to Altered Bladder Reflexes in the Rat,” Journal of Pharmacology and Experimental Therapeutics 323, no. 1 (2007): 227–235. 17636010 10.1124/jpet.107.125435

[40] M. Michishita , K. Yano , K. I. Tomita , O. Matsuzaki , and K. I. Kasahara , “Piezo1 Expression Increases in Rat Bladder After Partial Bladder Outlet Obstruction,” Life Sciences 166 (2016): 1–7. 27756599 10.1016/j.lfs.2016.10.017

[41] M. G. Dalghi , D. R. Clayton , W. G. Ruiz , et al., “Expression and Distribution of PIEZO1 in the Mouse Urinary Tract,” American Journal of Physiology. Renal Physiology 317, no. 2 (2019): F303–F321. 31166705 10.1152/ajprenal.00214.2019 PMC6732449

[42] H. Liu , P. Li , M. Zhao , et al., “Activation of Piezo1 Channels Enhances Spontaneous Contractions of Isolated Human Bladder Strips via Acetylcholine Release From the Mucosa,” European Journal of Pharmacology 983 (2024): 176954. 39237075 10.1016/j.ejphar.2024.176954

[43] B. R. Sharopov , I. B. Philyppov , S. I. Yeliashov , et al., “Contribution of Transient Receptor Potential Vanilloid 1 (TRPV1) Channel to Cholinergic Contraction of Rat Bladder Smooth Muscle,” Journal of Physiology 602, no. 15 (2024): 3693–3713. 38970617 10.1113/JP285514

