# Supplementary material for: Pharmacological Separation of Mechanosensory Mechanisms in Rat Urinary Bladder Ex Vivo
Source: Pharmacol Res Perspect. 2026 Apr 1;14(2):e70242. doi: 10.1002/prp2.70242 (PMC13042623; doi:10.1002/prp2.70242)
Supplement: Supplementary file 1 — Figure S1: Evaluation of the magnitude of the effect of drugs, specific for mechanosensitive ion channels, on the stress–strain relationship of rat bladder DSM strips. A–D: Results of the calculation the mean ± SD of the division of variable X (i.e., the stress values at a given strain in the presence of the drug) by variable Y (i.e., the control stress values at the same strain in the absence of the drug) for the TREK1 channel inhibitor, L‐methionine (A), the TRPV4 channel agonist, GSK1016790A (B), the TRPV4 channel inhibitor, HC‐067047 (C), and the PIEZO1 channel activator, Yoda1 (D), according to the formulas [Ruiz Espejo Mariano, 2015]: The data used to construct plots A–D are those presented in panels A, B of Figure 2, Figure 3 and Figure 4 of the main text. [file PRP2-14-e70242-s001.pdf]

Supporting Information

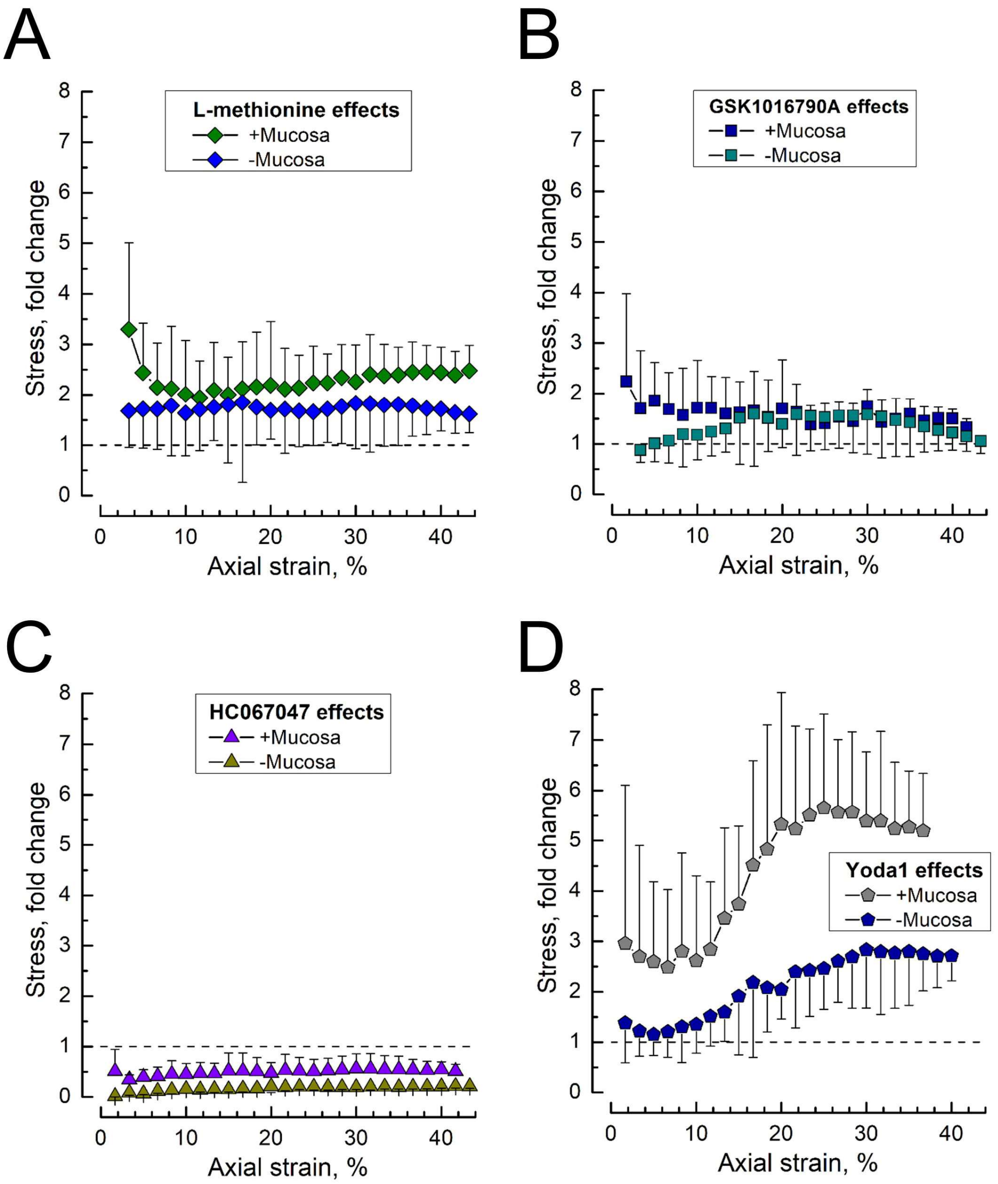

Supplementary Figure 1. Evaluation of the magnitude of the effect of drugs, specific for mechanosensitive ion channels, on the stress-strain relationship of rat bladder DSM strips.

A-D: Results of the calculation the mean±SD of the division of variable X (i.e., the stress values at a given strain in the presence of the drug) by variable Y (i.e., the control stress values at the same strain in the absence of the drug) for the TREK1 channel inhibitor, L-methionine (**A**), the TRPV4 channel agonist, GSK1016790A (**B**), the TRPV4 channel inhibitor, HC-067047 (**C**), and the PIEZO1 channel activator, Yoda1 (**D**), according to the formulas [Ruiz Espejo Mariano, 2015]:

$$\text{Mean:X/Y} = \text{Mean[X]} \times \text{Mean[1/Y]}$$

$$\text{SD:X/Y} = \sqrt{\text{Mean}[X^2] \times \text{Mean}[1/Y]^2 - (\text{Mean}[X] \times \cdot \text{Mean}[1/Y])^2}$$

The data used to construct plots **A-D** are those presented in panels **A, B** of Figure 2, Figure 3 and Figure 4 of the main text.

References

Ruiz Espejo Mariano (2015). How do I calculate the variance of the ratio of two independent variables? Retrieved on 2026-03-20 from: https://www.researchgate.net/post/How-do-I-calculate-the-variance-of-the-ratio-of-two-independent-variables/560e38296225ff79878b457c/citation/download.
